# Very Elderly Patients With Atrial Fibrillation Treated With Edoxaban: Impact of Frailty on Outcomes

**DOI:** 10.1016/j.jacadv.2023.100569

**Published:** 2023-08-24

**Authors:** Gentian Denas, Giacomo Zoppellaro, Serena Granziera, Leopoldo Pagliani, Franco Noventa, Sabino Iliceto, Vittorio Pengo

**Affiliations:** a Department of Cardiac, Thoracic, Vascular Sciences, and Public Health, Cardiology Clinic, University of Padua, Padua, Italy; b Cardiology Unit, High Specialization Rehabilitation Hospital, Treviso, Italy; c Ospedale Civile di Venezia, Azienda ULSS3 Serenissima, Venice, Italy; d Department of Physical and Rehabilitation Medicine, “Villa Salus” Hospital, Mestre, Italy; e University of Padua, Padua, Italy

**Keywords:** anticoagulation, atrial fibrillation, edoxaban, frailty, very elderly

## Abstract

**Background:**

Age and frailty are associated with underuse of anticoagulation in elderly patients with atrial fibrillation (AF).

**Objectives:**

This study aimed at assessing major clinical outcomes in very elderly patients with AF treated with recommended dose edoxaban and look for a possible relation with frailty measured by a validated score.

**Methods:**

This prospective multicenter cohort study enrolled consecutive very elderly (age ≥80 years) anticoagulation-naïve patients starting recommended doses of edoxaban. Upon entry into the study, patients were categorized into nonfrail, prefrail and frail with the SHARE-FI (Survey of Health, Ageing, and Retirement in Europe–Frailty Index) score. The primary outcome was a composite incidence of stroke/systemic embolism, major bleeding, clinically relevant nonmajor bleeding, and death between frail and fitter patients over 2 years follow-up. Secondary outcomes were frailty-related incidence of the individual components part of the composite outcome.

**Results:**

Of the 180 screened patients, 176 were enrolled in the study. Of these, 58 (32.9%) were frail, 35 (19.8%) prefrail, and 83 (47.2%) nonfrail. The composite outcome occurred in 49 patients (18.9% per patient-year). No difference in the primary endpoint between frail and fitter patients (incidence rate ratio: 1.2; 95% CI: 0.6-2.2) was observed. On multivariable analysis, anemia was significantly related to the primary outcome (HR: 3.6; 95% CI: 1.8-7.3; *P* < 0.001), while frailty was not (frail vs nonfrail HR: 0.9; 95% CI: 0.5-1.8). No difference across frailty categories of the individual components of composite events was observed, except for death.

**Conclusions:**

Anticoagulation with recommended dose edoxaban is feasible in very elderly patients with AF even if frail. (ESCAPE [Edoxaban and Frailty in Senior Individuals]; NCT03524924)

The risk of atrial fibrillation (AF) increases with advancing age and is associated with cardiovascular disease, risk factor burden, and frailty.^1^^,^^2^ AF significantly increases the risk of stroke in the presence of risk factors. The risk of stroke increases significantly with age, especially in the very elderly aged 80 years and above. Anticoagulation significantly reduces stroke risk, with the recommended doses of direct oral anticoagulants (DOACs) offering a better net clinical benefit in terms of lower incidence of stroke, death, and intracranial hemorrhage with no difference in major bleeding.^3^ Hence, very elderly patients would benefit most from anticoagulation but are paradoxically less likely treated with these drugs with one of the most common reasons for oral anticoagulation deter being frailty.^2^^,^^4^, ^5^, ^6^, ^7^ Frailty has not been specifically evaluated in DOAC registration trials and postmarketing observational cohorts. However, treatment with edoxaban provided an even greater absolute net clinical benefit in the elderly,^8^ and in vulnerable patient subgroups.^9^, ^10^, ^11^, ^12^, ^13^ Frailty is a complex condition generally attributed to age, but which in reality only partly overlaps with it. Given the lack of a universally accepted standardized definition of frailty, it leaves much to the subjective perception of the physician making the assessment.^14^ Indeed, cardiologists subjectively identify phenotypic frailty in the presence of problems with walking, cognition, malnutrition, sarcopenia, sex, and age.^14^^,^^15^ Several clinical frailty assessment tools have been developed but, due to complexity, are hardly used in the routine outpatient assessment of AF.^14^^,^^15^ One tool,^16^ SHARE-FI (Survey of Health, Ageing, and Retirement in Europe–Frailty Index), may be suitable for use in AF setting.^17^ Frailty plays an important role in AF management decision-making, and despite not being part of anticoagulation risk assessment scores, it impacts decision mostly leading to underdosing.^18^ Since frail very elderly patients are underrepresented in randomized controlled trials, evidence may not necessarily be translated to this population. Broad registry data coming from routine clinical practice show that edoxaban is initiated mainly in older patients, who are often anticoagulation-naïve patients with AF.^19^ Although DOACs have shown a clear net clinical outcome over warfarin in all age categories in registration randomized clinical trials,^3^ it is currently unclear whether these results can be replicated in patients with AF not meeting the inclusion/exclusion criteria of these trials, such as frail patients.

The aim of this study was to evaluate clinical outcomes in very elderly patients with AF treated with recommended doses of edoxaban and look for a possible relation with frailty as evaluated by a reproducible score.

## Methods

### Study population and intervention

ESCAPE (Edoxaban Performance in Senior Citizens With Nonvalvular Atrial Fibrillation Evaluated per Frailty) was a multicenter prospective cohort study enrolling consecutive patients of 80 years or older with a new diagnosis of AF not related to rheumatic mitral stenosis or patients with mechanical valves that were considered for oral anticoagulation with recommended doses of edoxaban according to the summary of product characteristics. The Ethics Committee of the Padua University Hospital approved the protocol. All participants gave written informed consent, and the study was performed in accordance with the principles of the Declaration of Helsinki. AF had to be diagnosed by means of an electrical tracing within the 30 days preceding enrollment. In case patients had started low molecular weight heparin or vitamin K antagonists, they were switched to edoxaban, according to the summary of product characteristics. Patients were excluded (exclusion criteria provided in the Supplemental Appendix) if they had a known contraindication to anticoagulation therapy in general or edoxaban in particular. Baseline demographic characteristics, comorbidities, stroke and bleeding risk factors, medications, and blood tests, including hemoglobin, creatinine, alanine transaminase, aspartate transaminase, were measured at baseline and reassessed at each follow-up visit. Stroke risk was assessed using the CHA_2_DS_2_VASc score.^20^ The first patient was enrolled in November 2018. Follow-up visits were set at 3, 6, 12, and 24 months. Creatinine clearance (CrCl) was calculated at each visit using the Cockcroft-Gault formula, and the recommended doses of edoxaban were administered to all patients. Low dose edoxaban regimen, 30 mg once daily, was prescribed in patients with moderate or severe renal impairment (CrCl 15-50 mL/min), in patients with body ≤60 kg, and in case of concomitant use of P-glycoprotein inhibitors such as ciclosporin, dronedarone, erythromycin, or ketoconazole. High dose edoxaban regimen, 60 mg once daily, was prescribed in the rest of patients. The off-label use of very low dose edoxaban, 15 mg, was not permitted. Edoxaban dosage (30 mg or 60 mg) could be changed according to CrCl, weight, or medications at each visit. Patients could be assessed at any time in between in case of adverse events, hospitalizations, or contacts requiring medical attention. At follow-up visits, the dedicated physician assessed adherence and compliance to therapy, relevant blood tests, and patient clinical status. Follow-up was conducted in person during medical office visits or by phone contact during the COVID-19 pandemic lockdown. The last patient was contacted on March 2022. The end of follow-up was considered the completion of 24 months of follow-up, the occurrence of an event part of the study outcome, permanent interruption of edoxaban, whichever came first. Temporary interruption of edoxaban for invasive procedures/interventions was permitted following the standard DOAC protocol.^1^^,^^2^^,^^21^

### Classification of patients according to frailty

Frailty was evaluated in each patient at baseline using the SHARE-FI score.^16^ This score has been widely validated and its use suggested^17^ as a simple and feasible tool to use in an ambulatory setting. SHARE-FI assesses frailty based on the following 5 variables: fatigue, loss of appetite, grip strength, functional difficulties, and physical activity. All patients underwent a face-to-face interview to answer a few multiple-choice questions. Handgrip strength was measured using a hand-grip dynamometer (KERN MAP 80K1S, KERN & Sohn GmbH). Answers were processed by an online calculator that categorized the patient as nonfrail, prefrail, or frail (an online frailty calculator can be found in Romero-Ortuno et al^16^ under electronic supplemental material). The questionnaire and the scores for each category are provided in the Supplemental Appendix. Frailty status was not used to drive edoxaban dosing.

### Outcome definition

The primary outcome was a composite of stroke/systemic embolism, major bleeding, clinically relevant nonmajor (CRNM) bleeding, and death. Primary endpoint was compared in frail vs fitter patients. The secondary outcomes were the frailty-related incidence of the primary outcome and of its individual components.

Stroke or systemic embolism was diagnosed by imaging. Major bleeding was defined according to the International Society on Thrombosis and Hemostasis.^22^ CRNM bleeding was defined as bleeding that did not meet the definition of major bleeding but required medical face-to-face evaluation^23^ or discontinuation of anticoagulation.

### Statistical analysis

The sample size was calculated based on the results of the ENGAGE AF-TIMI 48 (Effective Anticoagulation with Factor Xa Next Generation in Atrial Fibrillation–Thrombolysis In Myocardial Infarction 48) population.^8^^,^^24^

We calculated an overall annualized incidence of 20% based on the net composite outcome (stroke/systemic embolism, major bleeding, and death, 2.7%, 3.6%, and 4.8% respectively) in the *very elderly* ENGAGE AF-TIMI 48 population^8^ and CRNM bleeding approximated from the *overall* ENGAGE AF-TIMI 48 population^24^. We conducted a single sample estimate of precision based on an assumed event rate of 20% with a 2-sided 95.0% CI and a postulated confidence width of ±6%. The resulted sample size was 180 patients. Demographic and clinical characteristics of patients are reported by frequencies and proportions for categorical variables and mean, and standard deviation for continuous variables, as appropriate, and differences across frailty groups (frail, prefrail, and nonfrail) were assessed using 1-way analysis of variance or the chi-square test. Exposure time was calculated as the difference between the date of enrollment and the date of an outcome occurrence, discontinuation of therapy, the completion of 24 months of follow-up, or death, whichever came first. Time to a composite event was calculated as the time to the first event in the composite definition. For the outcomes, the proportion of the single events and the composite outcome were compared in the frail classes by chi-squared and chi-squared for trend statistics. The incidence rate ratio (IRR) was estimated between frail vs fitter (prefrail and nonfrail) for the events of interest. The cumulative proportion of primary composite outcome occurrence was estimated by the Kaplan-Meier method, and frailty groups were compared using the log-rank test. Cox proportional hazards analysis was used to assess predictors of the composite outcome by controlling for age, gender, and pre-existing conditions, and by introducing frailty in the model; clinical risk factors (anemia and chronic kidney disease) rather than biochemical factors (hemoglobin and CrCl) were introduced in the model. The proportional hazard assumption was tested by the Schoenfeld method. The threshold for statistical significance was a 2-sided *P* < 0.05.

## Results

**Figure 1:**
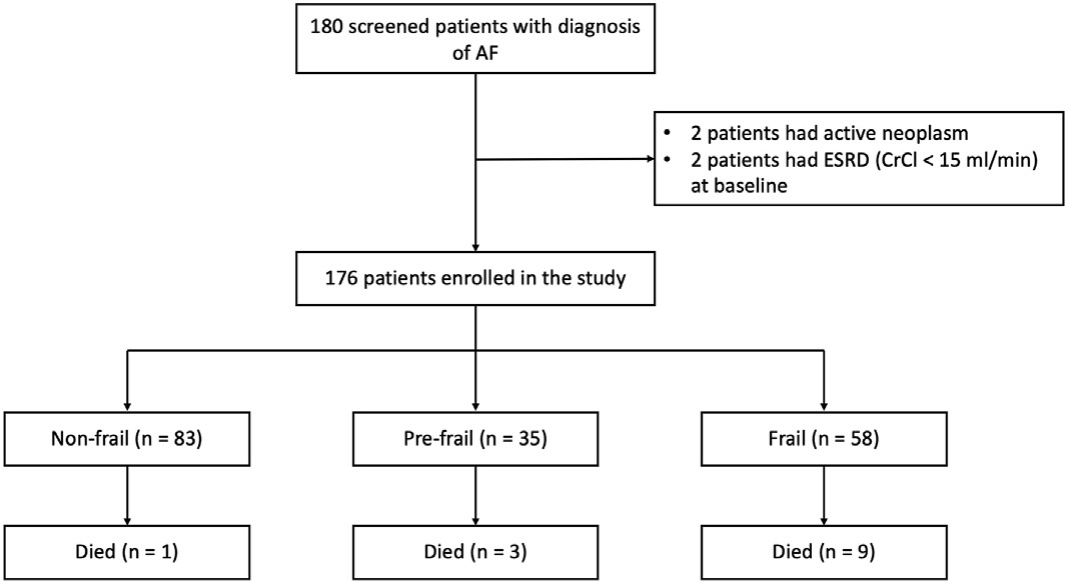
**Study Population** AF = atrial Fibrillation; CrCl = creatinine clearance; ESDR = end stage renal disease.

During the prespecified enrollment period, 180 patients with diagnosis of AF and aged 80 years or older were consecutively screened, and 176 of these patients were finally enrolled in the study (Figure 1). The baseline demographic and clinical characteristics of the study cohort are presented in Table 1. Overall, the mean age of the study cohort was 85 years (range 80-100 years), and 101 (57%) were female. Relevant comorbidities included hypertension, vascular disease, diabetes, heart failure, and previous stroke. The mean CHA_2_DS_2_VASc score was 4.4. Anemia was present in one-third of patients, while CrCl was below 50 ml/min in one-half of the patients. About 60% of patients met the dose reduction criteria and received edoxaban 30 mg once daily.

**Table 1:** Demographic and Baseline Clinical Characteristics of the Patients

	All (N = 176)	Nonfrail (n = 83)	Prefrail (n = 35)	Frail (n = 58)	*P*_trend_^a^
Age (y)	85.2 ± 3.9	84.2 ± 3.8	85.1 ± 3.4	86.5 ± 4.2	0.001
>85 y	73 (41.5)	27 (32.5)	16 (45.7)	30 (51.7)	0.021
Female	101 (57.3)	39 (46.9)	22 (62.8)	40 (68.9)	0.008
Weight (kg)	70.0 ± 12.0	70.7 ± 11.5	70.3 ± 11.5	68.9 ± 13.2	0.396
Body-mass index (kg/m^2^)	26.0 ± 3.6	25.5 ± 3.3	26.5 ± 3.8	26.4 ± 3.9	0.122
Creatinine clearance^b^	51.9 ± 17.5	54.4 ± 17.4	49.6 ± 15.6	49.9 ± 18.5	0.119
≤50 ml/min	89 (50.6)	37 (44.6)	21 (60.0)	31 (53.4)	0.257
<30 ml/min	8 (4.5)	0 (0.0)	0 (0.0)	8 (13.8)	<0.001
Hemoglobin (g/L)	131.5 ± 18.2	135.3 ± 17.5	130.4 ± 18.1	126.5 ± 18.3	0.002
AST (U/L)	24.6 ± 12.8	21.3 ± 7.4	26.7 ± 14.7	26.7 ± 12.8	0.398
ALT (U/L)	22.7 ± 17.0	21.4 ± 15.6	22.5 ± 12.2	24.5 ± 17.0	0.333
Hypertension	165 (93.8)	77 (92.8)	34 (97.1)	54 (93.1)	0.023
Congestive heart failure	21 (11.9)	7 (8.4)	3 (8.6)	11 (18.9)	0.07
Diabetes	28 (15.9)	11 (13.3)	5 (14.3)	12 (20.7)	0.245
Stroke	19 (10.8)	5 (6.0)	4 (11.4)	10 (17.2)	0.03
Vascular disease^c^	62 (35.2)	28 (33.7)	11 (31.4)	23 (39.7)	0.496
Anemia^d^	57 (32.3)	20 (24.1)	13 (37.1)	24 (41.4)	0.027
Chronic kidney disease^e^	89 (50.6)	37 (44.6)	21 (60.0)	31 (53.4)	0.257
CHA_2_DS_2_VASc score^f^	4.4 ± 1.1	4.1 ± 0.9	4.3 ± 0.9	4.8 ± 1.2	<0.001
CHA_2_DS_2_VASc ≥4	142 (80.7)	61 (73.5)	30 (85.7)	51 (87.9)	0.028
Edoxaban 30 mg	106 (60.2)	47 (56.6)	22 (62.8)	37 (63.7)	0.37

Values are mean ± SD or n (%).

AST = aspartate transaminase.

a *P* value represents 1-way analysis of variance comparison for continuous variables and Pearson chi-square for categorical variables.

b The mean creatinine clearance according to the Cockcroft-Gault equation.

c Anemia according to World Health Organization definition: hemoglobin <130 g/L in males and <120 g/L in female patients.

d Includes previous myocardial infarction.

e Chronic kidney disease is defined as creatinine clearance ≤50 ml/min.

f CHA_2_DS_2_-VASc score ranges from 0 to 9.

One-third (32.9%) of the patients were frail, 19.8% were prefrail, and 47.2% were nonfrail. Frail patients were more frequently female and older (mean age 86.5 years). Of the major chronic conditions, more frail patients had congestive heart failure, diabetes, a history of stroke, and anemia. This translated to a higher mean CHA_2_DS_2_VASc score among frail patients (4.8 in frail, 4.3 in prefrail, and 4.1 in nonfrail). More frail patients had a CrCl of ≤50 ml/min. Edoxaban 30 mg was administered in frail, prefrail, and nonfrail patients in 63.7%, 62.8%, and 56.6%, respectively (*P* = 0.6).

Patients were followed up for a total of 259 patient-years. No patients were lost to follow up. There were 12 patients that prematurely discontinued edoxaban (patient characteristics and reasons are listed in the Supplemental Appendix). Patients who prematurely discontinued edoxaban did so because of adverse events unrelated to endpoints, emerging concomitant comorbidities, or because they were no longer capable of participation due to terminal illness. During the study period, the primary composite outcome occurred in 49 patients (18.9% per patient-year, 95% CI: 14%-25%), 8 events were strokes, 12 were major bleeding, 16 were CRNM bleeding, and 13 were deaths. The IRR for the composite outcome (IRR: 1.2; 95% CI: 0.6-2.2), as well as for its individual components (Table 2) did not statistically differ between frail and fitter patients.

**Table 2:** Primary Composite Outcome and its Individual Components Across Frailty Groups

Frailty Categories	N	Stroke	Major Bleeding	CRNMB	Death	Composite Outcome (%; 95% CI)
Nonfrail	83	6 (7.2%)	6 (7.2%)	8 (9.6%)	1 (1.2%)	21 (25.3; 12.9-32.1)
Prefrail	35	0	3 (8.6%)	4 (11.4%)	3 (8.6%)	10 (28.6; 13.7-52.5)
Frail	58	2 (3.4%)	3 (5.2%)	4 (6.9%)	9 (15.5%)	18 (31.0; 20.6-43.8)
All	176	8 (4.5%)	12 (6.8%)	16 (9.1%)	13 (7.4%)	49 (27.8; 21.7-34.9)
Chi-square *P*		0.201	0.803	0.599	0.006	0.752
Chi-square *P*_trend_		0.241	0.661	0.608	0.001	0.451
Frail vs fitter, IRR (95% CI)	58/118	0.7 (0.07-3.7)	0.7 (0.1-2.7)	0.7 (0.2-2.2)	4.6 (1.3-20.3)	1.2 (0.6-2.2)

Values are n (%) unless otherwise indicated.

Chi-square = Pearson chi-square; CRNMB = clinically relevant non-major bleeding; IRR = incidence rate ratio.

On multivariable analysis (Table 3) only anemia was significantly related to the primary outcome (HR: 3.6; 95% CI: 1.8-7.3; *P* < 0.001), while frailty was not (frail vs nonfrail HR: 0.9; 95% CI: 0.5-1.8). In the Schoenfeld method to assess the Cox proportional hazards assumption, there were no obvious trends in the residual plots for anemia (*P* = 0.30). The composite outcome was not significantly different among patients receiving edoxaban 60 mg (22.9%) vs edoxaban 30 mg (18.9%; HR: 0.9; 95% CI: 0.5-1.6; *P* = 0.80), nor were stroke (edoxaban 60 mg 4.3% vs edoxaban 30 mg 4.7%; HR: 0.9; 95% CI: 0.2-3,7; *P* = 0.80), major bleeding (edoxaban 60 mg 7.1% vs edoxaban 30 mg 6.6%; HR: 1.0; 95% CI: 0.3-3.3; *P* = 0.90), or CRNM bleeding (edoxaban 60 mg 11.4% vs edoxaban 30 mg 7.5%; HR: 1.4; 95% CI: 0.5-3.8; *P* = 0.50).

**Table 3:** Cox Regression Analysis of the Composite Outcome Across Demographic and Baseline Clinical Characteristics^a^

	Composite Outcome		Multivariable HR (95% CI)
	No (n = 127, 72.2%)	Yes (n = 49, 27.8%)	
Age (y)	84.9 ± 3.8	85.8 ± 4.3	1.0 (0.9-1.1)
Female	74 (73.3)	27 (26.7)	0.8 (0.4-1.5)
Body mass index (kg/m^2^)	25.9 ± 3.8	26.0 ± 2.9	1.0 (0.9-1.1)
Creatinine clearance^b^	52.7 ± 17.3	50.0 ± 17.8	-
Hemoglobin (g/L)	133.8 ± 16.8	125.5 ± 20.4	-
Hypertension	119 (72.1)	46 (27.9)	0.9 (0.3-3.3)
Congestive heart failure	15 (71.4)	6 (28.6)	0.6 (0.3-1.6)
Diabetes	21 (75.0)	7 (25.0)	0.6 (0.3-1.5)
Stroke	14 (73.7)	5 (26.3)	0.7 (0.2-1.9)
Vascular disease^c^	42 (67.7)	20 (32.3)	1.1 (0.6-2.1)
Anemia^d^	32 (57.1)	24 (42.9)	3.6 (1.8-7.3)
Chronic kidney disease^e^	62 (69.7)	27 (30.3)	1.4 (0.4-4.8)
Edoxaban 30 mg daily	76 (71.7)	30 (28.3)	2.4 (0.7-8.4)
Frail	87 (68.5)	31 (63.3)	0.9 (0.5-1.8)

Values are mean ± SD or n (%) unless otherwise indicated.

a Clinical risk factors (anemia and chronic kidney disease) rather than biochemical factors (hemoglobin and creatinine clearance) were introduced in the model.

b The mean creatinine clearance according to the Cockcroft-Gault equation.

c Anemia according to World Health Organization definition: hemoglobin <130 g/L in males and <120 g/L in female patients.

d Includes previous myocardial infarction.

e Chronic kidney disease is defined as creatinine clearance ≤50 ml/min.

The mean hemoglobin levels in patients with events (Table 3) were 125 ± 20 g/L, with levels in the frail, prefrail and nonfrail 119 ± 14 g/L, 126 ± 17 g/L, and 130 ± 25 g/L, respectively. The mean hemoglobin level in patients with stroke/systemic embolism was 126 ± 19 g/L, major bleeding 123 ± 16 g/L, and CRNM bleeding 125 ± 23 g/L. In patients that died during follow up, the mean hemoglobin level was 118 ± 19 g/L.

**Figure 2:**
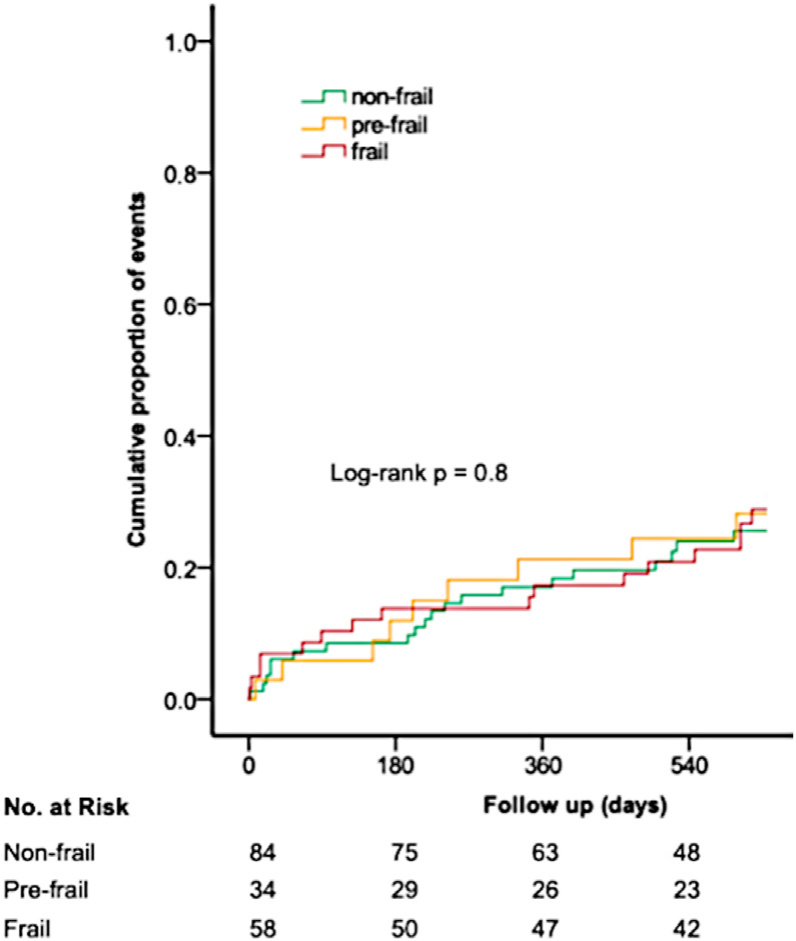
**Cumulative Incidence of the CompositeOutcome Across Frailty Categories**

The incidence of the composite outcome did not differ across frailty categories (20.3% per patient-year, 20.2% per patient-year, and 17.3% per patient-year in frail, prefrail, and no-frail patients, respectively; *P* = 0.80), and its cumulative proportion is illustrated in Figure 2. The annualized incidence of stroke/systemic embolism (2.3% vs 3.5%; *P* = 0.50), major bleeding (3.4% vs 5.3%; *P* = 0.40), or CRNM bleeding (3.4% vs 7.0%; *P* = 0.50) was similar for frail vs fitter patients.

There were 13 deaths (6.2% per patient-year) during the study period. Two were cardiovascular deaths. Eight patients died of lung disease and pneumonia, leading to respiratory failure, of these 2 due to COVID-19. Three deaths were due to neoplastic disease. Overall, mortality was higher among the frail (11.3% per patient-year vs 3.5% per patient-year, *P* = 0.019).

## Discussion

In this prospective cohort study, we assessed the clinical outcomes in very elderly patients with a new diagnosis of AF and starting anticoagulation therapy with recommended doses of edoxaban. Composite outcomes were not different between frail and fitter patients (prefrail and nonfrail) and across frailty categories (frail, pre-frail and non-frail). Although not powered to assess the differences among the 3 frailty groups, in our frail patients the incidence of events was low and of the same order of magnitude as that reported in other subgroup analysis^8^, ^9^, ^10^, ^11^, ^12^, ^13^ of the ENGAGE AF-TIMI 48,^24^ population. These analysis showed that edoxaban maintained the net clinical outcomes relative to warfarin despite age,^8^ frailty,^12^ burden of concomitant disease,^10^ risk of falling,^9^ stroke risk,^13^ and other factors conferring high risk of adverse events.^11^ Increasing age, frailty and factors associated with it, all confer an increased baseline risk of adverse events despite therapy.^8^, ^9^, ^10^, ^11^, ^12^, ^13^ However, we found that the composite outcome was similar across frailty groups when patients were treated with recommended doses of edoxaban (Central Illustration). Besides, we found that frail patients benefited the most in terms of stroke prevention, and this is in line with the findings that edoxaban provides incrementally larger absolute reductions in outcomes over warfarin in patients with higher CHA_2_DS_2_VASc scores.^13^

**Central Illustration:**
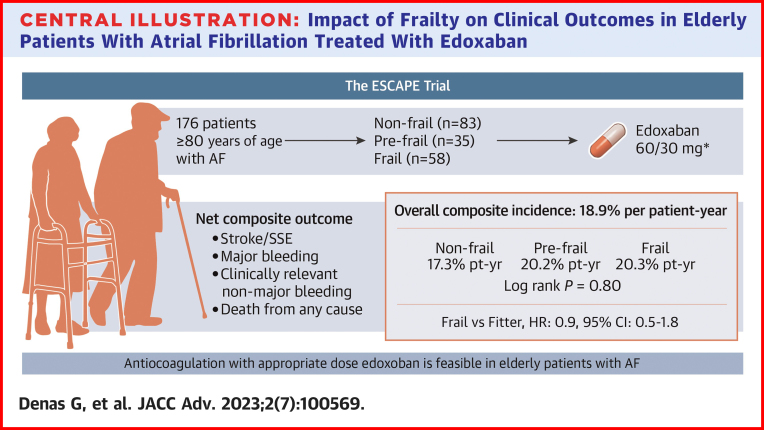
**Impact of Frailty on ClinicalOutcomes in Elderly Patients With Atrial Fibrillation Treated With Edoxaban**

Our results add to the data from the literature^8^, ^9^, ^10^, ^11^, ^12^, ^13^ that showed that older patients even if frail have more favorable outcomes with edoxaban. Frail and prefrail states are common with advancing age and raise concerns regarding the risk-benefit of oral anticoagulation therapy leading to underuse of anticoagulation.^21^ The appropriate anticoagulation management in the very elderly frail patients with AF represents an important clinical challenge, starting from the frailty definition. To avoid the phenotypic perception of frailty, we chose to measure it using SHARE-FI tool.^17^ Frailty is complex syndrome and no single assessment instrument performs the best for all settings and outcomes.^25^ With respect to other methods that assessed surrogates of frailty in post hoc analysis of ENGAGE AF-TIMI 48 trial,^9^^,^^10^^,^^12^ SHARE-FI is validated, reproducible, and with predictive validity.^25^^,^^26^ It should be noted that SHARE-FI has a reliance on self-report, with answers to the fatigue and appetite items may be unreliable in patients with cognitive impairment. However, we collected data during ambulatory visits with the help of family members and caregivers where possible.

The benefits of the recommended doses of DOACs over warfarin are consistent across the entire continuous spectrum of age,^3^ but whether these results can be replicated in frail patients that were underrepresented in clinical trials, is still to be elucidated.^27^ Available data come from post hoc analysis of the ENGAGE population not designed for the analysis undertaken.^9^^,^^12^ Frailty was not assessed using essential criteria for frailty definition such as weight loss, exhaustion, weakness, or slowness.^9^^,^^12^ Our trial was designed with a prespecified analysis on frailty, measured at baseline, and had a fair representation (one-third) of frail patients. Only 1 recent randomized controlled trial^28^ of elderly Japanese patients had a prespecified frailty analysis. With that respect, we found fewer stroke/systemic embolism events, but more major bleeding events (Supplemental Table 2) probably due to the use of the higher approved doses of edoxaban (60/30 mg vs 15 mg). However, the trial design, edoxaban dosage, and trial population were different from ours in terms of (but not limited to) contraindication to anticoagulation and ethnicity making comparisons arguable.^6^^,^^29^ Lower dose, 15 mg edoxaban, is not approved for clinical use in patients with AF, but might have a role in certain patients, especially those not deemed suitable for anticoagulation;^28^ however, the risk is that this might be generalized to patients with perceived frailty leading to worse outcomes. In our study, the composite outcome was not different among patients receiving edoxaban 60 mg vs edoxaban 30 mg, nor were stroke, major bleeding, or CRNM bleeding. Our results show that frailty alone should not be used as a dose reduction criterion. Rather, we found that only anemia at baseline was related to the incidence of the composite outcome. We, like others,^30^^,^^31^ found anemia to be associated with an increased risk of thromboembolic events, bleeding complications and mortality in anticoagulated patients with AF. Anemia is a potentially modifiable bleeding risk factor, thus, in very elderly frail patients, efforts should be made to identify and treat causes of anemia, and a cautious patient monitoring,^21^ rather than dose adjustment or discontinuation of anticoagulation.

Our data, likewise other reports,^8^, ^9^, ^10^^,^^12^ confirm that mortality is higher among vulnerable patients. While we cannot draw direct conclusions on the effect of anticoagulation on mortality since we assessed only anticoagulated patients. Death rates were even higher in nonanticoagulated patients.^28^

Our study has some strengths, such as the prospective design, enrollment of consecutive of very elderly frail patients with recent onset AF, a significant number of very elderly women (typically underrepresented in clinical trials), a standardized and reproducible frailty score. Furthermore, frailty status was not used as a criterion for dose reduction of edoxaban.

To our knowledge, this is the first study to have prospectively assessed frailty, with a validated and reproducible method in very elderly patients starting anticoagulation with edoxaban. Our findings suggest that edoxaban can be safely and effectively used in frail very elderly patients with AF, and that frailty should not be used as a deterrent for anticoagulation.

### Study Limitations

The number of the outcomes was low and this resulted in wide CIs. Frailty was assessed at baseline and during a long follow up some patients might have switched categories. Because different tools rely on the identification of different items, we could not perform a sensitivity analysis using another frailty tool. The study is not powered to assess differences between the 3 frailty groups. Finally, CRNM bleeding may carry a lower weight than major bleeding, however, it is of clinical importance as it may lead to treatment interruption, inappropriate dosing and worsen the patients’ quality of life.

## Conclusions

Anticoagulation with recommended doses of edoxaban is feasible in very elderly patients with AF even if frail.

PERSPECTIVES **COMPETENCY IN MEDICAL KNOWLEDGE:** AF is the most common arrhythmia with increasing age. AF has an increased risk of stroke especially in very elderly frail patients.

**COMPETENCY IN PATIENT CARE:** Stroke prevention in AF includes the use of anticoagulant drugs. However, in the very elderly (and frail) patients these drugs are underused because of fear of adverse events.

**TRANSLATIONAL OUTLOOK 1:** In the present study we assessed the incidence of major outcomes in very elderly (≥80 years) with AF starting anticoagulation with edoxaban.

**TRANSLATIONAL OUTLOOK 2:** Recommended dose edoxaban was feasible in very elderly patients with AF even if frail.

## Funding support and author disclosures

Nonconditional support from Daiichi Sankyo Italy was received. The authors have reported that they have no relationships relevant to the contents of this paper to disclose.

## References

[1] January C.T., Wann L.S., Calkins H. (2019). 2019 AHA/ACC/HRS focused update of the 2014 AHA/ACC/HRS guideline for the management of patients with atrial fibrillation: a report of the American College of Cardiology/American Heart Association Task Force on Clinical Practice Guidelines and the Heart Rhythm Society. J Am Coll Cardiol.

[2] Hindricks G., Potpara T., Dagres N. (2021). 2020 ESC guidelines for the diagnosis and management of atrial fibrillation developed in collaboration with the European Association for Cardio-Thoracic Surgery (EACTS): the TASK Force for the Diagnosis and Management of Atrial Fibrillation of the European Society of Cardiology (ESC) developed with the special contribution of the European Heart Rhythm Association (EHRA) of the ESC. Eur Heart J.

[3] Carnicelli A.P., Hong H., Connolly S.J. (2022). Direct oral anticoagulants versus warfarin in patients with atrial fibrillation: patient-level network meta-analyses of randomized clinical trials with Interaction testing by age and sex. Circulation.

[4] Ko D., Lin K.J., Bessette L.G. (2022). Trends in use of oral anticoagulants in older Adults with newly diagnosed atrial fibrillation, 2010-2020. JAMA Netw Open.

[5] Volgman A.S., Nair G., Lyubarova R. (2022). Management of atrial fibrillation in patients 75 Years and older: JACC state-of-the-art review. J Am Coll Cardiol.

[6] Giugliano R.P. (2022). Non-vitamin K antagonist oral anticoagulants in older and frail patients with atrial fibrillation. Eur Heart J Suppl.

[7] Wojszel Z.B., Kasiukiewicz A. (2020). Determinants of anticoagulant therapy in atrial fibrillation at discharge from a geriatric ward: cross sectional study. J Thromb Thrombolysis.

[8] Kato E.T., Giugliano R.P., Ruff C.T. (2016). Efficacy and safety of edoxaban in elderly patients with atrial fibrillation in the ENGAGE AF-TIMI 48 trial. J Am Heart Assoc.

[9] Steffel J., Giugliano R.P., Braunwald E. (2016). Edoxaban versus warfarin in atrial fibrillation patients at risk of falling: ENGAGE AF-TIMI 48 analysis. J Am Coll Cardiol.

[10] Nicolau A.M., Corbalan R., Nicolau J.C. (2020). Efficacy and safety of edoxaban compared with warfarin according to the burden of diseases in patients with atrial fibrillation: insights from the ENGAGE AF-TIMI 48 trial. Eur Heart J Cardiovasc Pharmacother.

[11] Gencer B., Eisen A., Berger D. (2022). Edoxaban versus Warfarin in high-risk patients with atrial fibrillation: a comprehensive analysis of high-risk subgroups. Am Heart J.

[12] Wilkinson C., Wu J., Searle S.D. (2020). Clinical outcomes in patients with atrial fibrillation and frailty: insights from the ENGAGE AF-TIMI 48 trial. BMC Med.

[13] de Groot J.R., Ruff C.T., Murphy S.A. (2021). Edoxaban versus warfarin in patients with atrial fibrillation in relation to the risk of stroke: a secondary analysis of the ENGAGE AF-TIMI 48 study. Am Heart J.

[14] Diemberger I., Fumagalli S., Mazzone A.M. (2022). Perceived vs. objective frailty in patients with atrial fibrillation and impact on anticoagulant dosing: an ETNA-AF-Europe sub-analysis. Europace.

[15] Fumagalli S., Potpara T.S., Bjerregaard Larsen T. (2017). Frailty syndrome: an emerging clinical problem in the everyday management of clinical arrhythmias. The results of the European Heart Rhythm Association survey. Europace.

[16] Romero-Ortuno R., Walsh C.D., Lawlor B.A., Kenny R.A. (2010). A frailty instrument for primary care: findings from the Survey of Health, Ageing and Retirement in Europe (SHARE). BMC Geriatr.

[17] Heidbuchel H., Verhamme P., Alings M. (2015). Updated European Heart Rhythm Association Practical Guide on the use of non-vitamin K antagonist anticoagulants in patients with non-valvular atrial fibrillation. Europace.

[18] Steinberg B.A., Shrader P., Thomas L. (2016). Off-label dosing of non-vitamin K antagonist oral anticoagulants and adverse outcomes: the ORBIT-AF II registry. J Am Coll Cardiol.

[19] De Caterina R., Kelly P., Monteiro P. (2019). Characteristics of patients initiated on edoxaban in Europe: baseline data from edoxaban treatment in routine clinical practice for patients with atrial fibrillation (AF) in Europe (ETNA-AF-Europe). BMC Cardiovasc Disord.

[20] Friberg L., Rosenqvist M., Lip G.Y. (2012). Evaluation of risk stratification schemes for ischaemic stroke and bleeding in 182 678 patients with atrial fibrillation: the Swedish Atrial Fibrillation cohort study. Eur Heart J.

[21] Steffel J., Collins R., Antz M. (2021). 2021 European Heart Rhythm Association practical guide on the use of non-vitamin K antagonist oral anticoagulants in patients with atrial fibrillation. Europace.

[22] Schulman S., Kearon C. (2005). Subcommittee on control of anticoagulation of the S, Standardization Committee of the International Society on T, Haemostasis. Definition of major bleeding in clinical investigations of antihemostatic medicinal products in non-surgical patients. J Thromb Haemost.

[23] Kaatz S., Ahmad D., Spyropoulos A.C., Schulman S., Subcommittee on Control of Anticoagulation (2015). Definition of clinically relevant non-major bleeding in studies of anticoagulants in atrial fibrillation and venous thromboembolic disease in non-surgical patients: communication from the SSC of the ISTH. J Thromb Haemost.

[24] Giugliano R.P., Ruff C.T., Braunwald E. (2013). Edoxaban versus warfarin in patients with atrial fibrillation. N Engl J Med.

[25] Oviedo-Briones M., Rodriguez-Laso A., Carnicero J.A. (2022). The ability of eight frailty instruments to identify adverse outcomes across different settings: the FRAILTOOLS project. J Cachexia Sarcopenia Muscle.

[26] Oviedo-Briones M., Laso A.R., Carnicero J.A. (2021). A comparison of frailty assessment instruments in different clinical and Social care settings: the Frailtools project. J Am Med Dir Assoc.

[27] Goto S., Goto S. (2022). A patient-level meta-analysis: the end of the era of direct oral anticoagulant developmental trials in patients with atrial fibrillation?. Circulation.

[28] Okumura K., Akao M., Yoshida T. (2020). Low-dose edoxaban in very elderly patients with atrial fibrillation. N Engl J Med.

[29] Chiang C.E., Wang K.L., Lin S.J. (2015). Asian strategy for stroke prevention in atrial fibrillation. Europace.

[30] Al-Hussainy N., Kragholm K.H., Lundbye-Christensen S. (2022). Safety and efficacy of direct oral anticoagulants in patients with anaemia and atrial fibrillation: an observational nationwide Danish cohort study. Eur Heart J Qual Care Clin Outcomes.

[31] Westenbrink B.D., Alings M., Connolly S.J. (2015). Anemia predicts thromboembolic events, bleeding complications and mortality in patients with atrial fibrillation: insights from the RE-LY trial. J Thromb Haemost.

