# Supplementary material for: Very Elderly Patients With Atrial Fibrillation Treated With Edoxaban: Impact of Frailty on Outcomes
Source: JACC Adv. 2023 Aug 24;2(7):100569. doi: 10.1016/j.jacadv.2023.100569 (PMC11198574; doi:10.1016/j.jacadv.2023.100569)
Supplement: Supplementary data [file mmc1.docx]

**SUPPLEMENTAL APPENDIX**

# **Exclusion criteria**

1. Concomitant use of other anticoagulants at baseline
2. End-stage renal disease (CrCl < 15 mL/min) or on dialysis
3. Severe hepatic impairment
4. Elevated liver enzymes (ALT/AST > 2 x ULN) or total bilirubin ≥ 1.5 x ULN at baseline
5. Recent (within 1 month) or persisting gastrointestinal bleeding;
6. Known or suspected esophageal varices
7. Active neoplasm
8. Diagnosis of arteriovenous malformations, vascular aneurysms or major intraspinal or intracerebral vascular abnormalities
9. Life expectancy <1 year
10. Concomitant use of strong P-glycoprotein (P-gp) inhibitors other than verapamil, quinidine, or dronedarone
11. Clinically significant active bleeding or high risk of bleeding conditions such as: recent brain or spinal injury; recent brain, spinal or ophthalmic surgery; recent intracranial hemorrhage
12. Known contraindications or hypersensitivity to the active substance or to any of the excipients of edoxaban

# **Switching to and from Edoxaban to other anticoagulant treatment**

1. Switching from Vitamin K antagonist (VKA) to Edoxaban required the discontinuation of the VKA and starting of edoxaban when the international normalized ratio (INR) was ≤ 2.5.
2. Switching from subcutaneous low molecular weight heparin (LMWH) or fondaparinux to Edoxaban required the discontinuation of subcutaneous anticoagulant and the start of edoxaban at the time of the next scheduled subcutaneous anticoagulant dose. Edoxaban and subcutaneous anticoagulants (or intravenous unfractionated heparin (UFH)) should not be administered simultaneously.

# **SHARE-FI calculation**

## **Questionnaire**

1. Exhaustion was identified as a positive response to the question: "In the last month, have you had too little energy to do the things you wanted to do?". A positive answer (Yes) was re-coded as 1, and No was re-coded as 0.
2. The weight loss criterion was fulfilled by reporting a "Diminution in desire for food" in response to the question: "What has your appetite been like?" or, in the case of a non-specific or uncodeable response to this question, by responding "Less" to the question: "So, have you been eating more or less than usual?". The presence of the criterion was coded as 1 and its absence as 0.
3. Weakness was assessed by handgrip strength (Kg) using a dynamometer (KERN MAP 80K1S, KERN & Sohn GmbH, Balingen, Germany). Two consecutive measurements were taken from the left and right hands. The highest of the four was selected. This variable was kept continuous.
4. Slowness was defined as a positive answer to either of the following two items: "Because of a health problem, do you have difficulty [expected to last more than 3 months] walking 100 metres?" or "... climbing one flight of stairs without resting?". One or two positive answers received the score of 1, and two negative answers received the score of 0.
5. The low activity criterion was assessed by the question: "How often do you engage in activities that require a low or moderate level of energy such as gardening, cleaning the car, or doing a walk?". This variable was kept ordinal: 1 = "More than once a week"; 2 = "Once a week"; 3 = One to three times a month

## **Calculation and results**

|  | Predicted D-Factor Score | |
| --- | --- | --- |
|  | **Females** | **Males** |
| **NON-FRAIL** | < 0.3151361243 | < 1.211878526 |
| **PRE-FRAIL** | < 2.1301121973 | < 3.0052612772 |
| **FRAIL** | < 6 | < 7 |

# **Supplemental Table 1. Patient characteristics and reasons for premature edoxaban termination**

| **Patient** | **Gender** | **Age** | **Edoxaban dose mg** | **CHADSVASc** | **Frailty category** | **Reasons for premature termination of Edoxaban therapy** |
| --- | --- | --- | --- | --- | --- | --- |
| #1 | M | 85 | 30 | 4 | Pre frail | Terminated early by the cardiologist after successful electrical cardioversion |
| #2 | F | 86 | 30 | 3 | Non frail | Decided to stop therapy in the early days after initiation for headache |
| #3 | F | 91 | 30 | 4 | Pre frail | Switch to VKA for GFR deterioration below 15ml/min |
| #4 | F | 92 | 30 | 4 | Frail | Switch to VKA for GFR deterioration below 15ml/min |
| #5 | F | 82 | 60 | 4 | Non frail | Switch to VKA by consulting hematologist for polycythemia vera |
| #6 | M | 86 | 30 | 2 | Non frail | Terminated for cerebral neoplastic disease |
| #7 | M | 81 | 30 | 5 | Non frail | Switch to VKA for TAVI |
| #8 | M | 82 | 30 | 3 | Non frail | Terminated therapy for alcohol abuse |
| #9 | F | 82 | 30 | 6 | Pre frail | Switch to another DOAC for skin rash |
| #10 | F | 83 | 30 | 5 | Pre frail | Switch to VKA for GFR deterioration below 15ml/min |
| #11 | M | 83 | 60 | 3 | Frail | Terminal illness |
| #12 | M | 81 | 60 | 5 | Non frail | Terminated for neoplastic disease |

# **Supplemental Table 2. Comparison of ESCAPE and ELDERCARE trial results**

|  | ESCAPE  Event Rates (%/pt-y) | ELDERCARE  Event Rates (%/pt-y)* |
| --- | --- | --- |
| Stroke/systemic embolism | 1.8 | 2.3 |
| Major bleeding | 3.7 | 3.3 |
| CRNM bleeding | 6.2 | 14.5 |
| All-cause mortality | 6.2 | 9.9 |

*event rates in patients receiving edoxaban 15 mg.
